# Physical Activity, Sedentary Behavior, and Sleep on Twitter: Multicountry and Fully Labeled Public Data Set for Digital Public Health Surveillance Research

**DOI:** 10.2196/32355

**Published:** 2022-02-14

**Authors:** Zahra Shakeri Hossein Abad, Gregory P Butler, Wendy Thompson, Joon Lee

**Affiliations:** 1 Department of Biomedical Informatics Harvard Medical School Harvard University Boston, MA United States; 2 Data Intelligence for Health Lab Cumming School of Medicine University of Calgary Calgary, AB Canada; 3 Centre for Surveillance and Applied Research Public Health Agency of Canada Ottawa, ON Canada; 4 Department of Community Health Sciences Cumming School of Medicine University of Calgary Calgary, AB Canada; 5 Department of Cardiac Sciences Cumming School of Medicine University of Calgary Calgary, AB Canada

**Keywords:** digital public health surveillance, social media analysis, physical activity, sedentary behavior, sleep, machine learning, online health information, infodemiology, public health database

## Abstract

**Background:**

Advances in automated data processing and machine learning (ML) models, together with the unprecedented growth in the number of social media users who publicly share and discuss health-related information, have made public health surveillance (PHS) one of the long-lasting social media applications. However, the existing PHS systems feeding on social media data have not been widely deployed in national surveillance systems, which appears to stem from the lack of practitioners and the public’s trust in social media data. More robust and reliable data sets over which supervised ML models can be trained and tested reliably is a significant step toward overcoming this hurdle. The health implications of daily behaviors (physical activity, sedentary behavior, and sleep [PASS]), as an evergreen topic in PHS, are widely studied through traditional data sources such as surveillance surveys and administrative databases, which are often several months out-of-date by the time they are used, costly to collect, and thus limited in quantity and coverage.

**Objective:**

The main objective of this study is to present a large-scale, multicountry, longitudinal, and fully labeled data set to enable and support digital PASS surveillance research in PHS. To support high-quality surveillance research using our data set, we have conducted further analysis on the data set to supplement it with additional PHS-related metadata.

**Methods:**

We collected the data of this study from Twitter using the Twitter livestream application programming interface between November 28, 2018, and June 19, 2020. To obtain PASS-related tweets for manual annotation, we iteratively used regular expressions, unsupervised natural language processing, domain-specific ontologies, and linguistic analysis. We used Amazon Mechanical Turk to label the collected data to self-reported PASS categories and implemented a quality control pipeline to monitor and manage the validity of crowd-generated labels. Moreover, we used ML, latent semantic analysis, linguistic analysis, and label inference analysis to validate the different components of the data set.

**Results:**

LPHEADA (Labelled Digital Public Health Dataset) contains 366,405 crowd-generated labels (3 labels per tweet) for 122,135 PASS-related tweets that originated in Australia, Canada, the United Kingdom, or the United States, labeled by 708 unique annotators on Amazon Mechanical Turk. In addition to crowd-generated labels, LPHEADA provides details about the three critical components of any PHS system: place, time, and demographics (ie, gender and age range) associated with each tweet.

**Conclusions:**

Publicly available data sets for digital PASS surveillance are usually isolated and only provide labels for small subsets of the data. We believe that the novelty and comprehensiveness of the data set provided in this study will help develop, evaluate, and deploy digital PASS surveillance systems. LPHEADA will be an invaluable resource for both public health researchers and practitioners.

## Introduction

### Digital Public Health Surveillance

Almost two-thirds of the world’s population now uses the internet, taking the global total to 4.57 billion (59%) by July 2020 [1]. Overall, 87% of internet users and 65% (3.96 billion) of the world’s total eligible population (ie, aged >13 years) now use social media. The combined time that these users spend on social media adds up to more than 1 million years every day [1], contributing to a large amount of user-generated data on different social media platforms. In 2020, Twitter alone reported 500 million tweets generated per day from 145 million daily active users. The low-cost data stream available on social media and other internet-based sources serves to makes research advances on digital public health surveillance (DPHS) more accessible for public health officials, clinicians, patients, and the public. This helps disseminate insights into different aspects of public health and promote healthy lifestyles and health policies [2,3]. The open access to the public data about users and their opinions, the ease of use, and a large user base have made Twitter one of the most popular data sources for studying different aspects of public health [4,5], with Google Scholar indexing 1.32 million articles mentioning Twitter and public health. Moreover, more than 85% of Twitter users, with a wide breadth of demographic groups [4], also use Facebook, Instagram, and YouTube (this number for other platforms varies between 52% and 82%) [1], indicating that Twitter users reasonably represent active social media users in general.

Since 2011, Twitter has been the most popular form of social media used for public health communication [6,7]. A recent scoping review of 755 articles on DPHS shows that Twitter is the most studied of all platforms and most used platform to study communicable diseases, behavioral risk factors, mental health, drug use, and vaccine [7].

### Limitations of Digital Public Health Data

However, a number of limitations that mainly stem from the limitations associated with the data are still the major obstacles toward the adoption of digital data for public health surveillance (PHS) [4,7]. Given the main aims of any PHS system are to measure, monitor, and improve the overall health status of their target populations, the systematic incorporation of time, demographics (ie, age and gender), and place data into the surveillance process is critical to the reliability and generalizability of this process [8,9]. However, nearly one-third (32%) of the DPHS studies published between 2005 and 2020 (with the majority of them related to behavioral risk factors surveillance) did not capture age, gender, or place information for their analyses [7]. Moreover, most studies on DPHS do not consider whether their findings are associated with the user’s personal experience (self-reported or not), leading to content bias, incorrect results, and potentially flawed interpretations [7].

Considering the location-dependent nature of health policies, along with the essential role of place data in assessing the representativeness of a PHS system, the impact of a PHS system can vary considerably with geographical location [10-13]. However, the number of DPHS studies that have stratified their results by a more granular geographic region is small [7]. Because of a lack of annotated data sets for the development of automatic models, more than two-thirds (69%) of DPHS studies published before 2020 are limited by labor-intensive, manual, and abstract analysis methods (eg, manual coding, qualitative analysis, and rule-based natural language processing [NLP]), which makes these studies limited in terms of sample size, scope, and generalizability [7].

Given that all of these challenges are data-oriented, an increase in both data quality and quantity enriched with concrete demographics and location information can help deal with all these challenges. Moreover, to facilitate the development and evaluation of robust machine learning (ML) models to address the limited scope of manual data analysis techniques, annotated data sets for various PHS aspects are required. However, only a handful of annotated data sets are publicly available for research on DPHS [14-21]. Jimeno-Yepes et al [15] provided an annotated data set of 1300 tweets related to disease symptoms and pharmacologic substances. The open data set developed by Aphinyanaphongs et al [16] contains 13,146 labeled tweets resulting from hashtag filtering and covers a time span from January 2010 to January 2015. This data set is developed for training binary classifiers to detect tweets that indicate e-cigarette use for smoking cessation. Crowdbreaks [18], an open health tracking platform, crowdsources the labeling of vaccine sentiment and COVID-19–related tweets to the public. Although the data set provided by this system, compared with other open DPHS data sets, is in a better position in terms of size, it lacks demographics and geospatial data, and each tweet is labeled by only 1 annotator (without any control over their labeling quality).

### Objective

Given that in addition to physical inactivity, as the leading risk factor for noncommunicable diseases and premature death [22], prolonged sedentary behavior and inadequate sleep are also important risk factors for chronic diseases [23], this work presents a multicountry and fully labeled digital public health data set (LPHEADA [Labelled Public Health Dataset]) of tweets related to physical activity, sedentary behavior, and sleep (PASS) that originated in Australia, Canada, the United Kingdom, or the United States. We selected these countries because they have some of the highest proportions of social media users in the world (Australia, 71%; Canada, 66%; the United Kingdom, 66%; the United States, 69%; and world, 51%) [1]. LPHEADA comprises 366,405 labels, labeled by 708 unique annotators on Amazon Mechanical Turk (AMT), for 122,135 unique tweets generated by 72,749 unique users between November 28, 2018, and June 19, 2020. AMT is a software service operated by Amazon that allows users (ie, requesters) to crowdsource work, broken into microtasks called human intelligence tasks (HITs), to a large number of workers who are compensated for each HIT completed [24]. As LPHEADA was collected and labeled in collaboration with the Public Health Agency of Canada to develop PASS indicators for the Canadian population, 80.83% (98,722/122,136) of the tweets included in our data set were collected from Canada. Tweets from the United States and the United Kingdom make up 8.35% (10,193/122,136) and 7.49% (9154/122,136) of the data set, respectively, and Australian tweets make up the remaining 3.33% (4067/122,136) of the data set.

Along with the labeled tweets, we provide detailed information on users’ gender, age range, and geospatial information, whether the tweet was self-reported, and whether it was posted by an organization. We evaluated the quality of the data set and its labels using latent semantic analysis, linguistic analysis, ML models, and truth inference models. The data set we provide in this paper can be used to develop unsupervised or supervised ML models for digital PASS surveillance.

## Methods

### Collection and Preparation of the Data Set

We collected the data of this study from Twitter using the Twitter livestream application programming interface (API) between November 28, 2018, and June 19*,* 2020. The entire data set (ie, 1,902,980,841 tweets) was filtered for Canadian tweets potentially relevant to PASS. From 22,729,110 collected Canadian tweets, 0.46% (103,911/22,729,110) were selected using keywords and regular expressions related to PASS categories. To define the search strings and regular expressions, we used NLP techniques (eg, topic modeling, language modeling, and linguistic analysis) to detect latent word patterns relevant to PASS-related contexts. Moreover, we pilot-tested the labeling process first to validate the extracted keywords and iteratively updated the list of keywords for each category after manually reviewing the labels and the filtered tweets. Multimedia Appendix 1 provides a complete list of the words used for generating regular expressions and filtering the data set. Each of these 103,911 tweets was labeled by 3 AMT workers, from which 95.01% (98,722/103,911) of tweets received 3 valid labels (ie, multiple or missing labels were invalid and rejected), with almost half of them related to physical activity. For the Canadian data set, 610 unique workers participated in our data labeling tasks and completed 103,911 HITs, from which 4.99% (5189/103,911) HITs were removed as they did not receive 3 valid answers. The majority of these workers (530/610, 86.9%) completed less than 100 HITs each, among which 30.9% (164/530) completed only 1 HIT each. Among all 610 workers, 1 (0.2%) worker completed 21,801 HITs, and 3 (0.5%) workers completed between 5000 and 10,000 HITs.

In addition to the Canadian tweets, we filtered a random subset of the data set for tweets that originated in the United Kingdom, the United States, and Australia. This data set spans the same data collection period as the Canadian data set and contains 70,239 labels collected for 23,413 tweets (ie, 3 labels per tweet). Adding the data from these countries will provide an important epidemiological diversity that can be used for implementing comparative studies and evaluating the generalizability of the PASS surveillance models trained on the Canadian data set.

### Labeling Process

We implemented a pipeline to create the crowdsourcing tasks, referred to as HITs by AMT, post them on AMT, collect the labels through a quality-check process, approve or reject the HITs, and store the results. To minimize noisy and low-quality data, we added a qualification requirement to our tasks and granted the labeling access to workers who had demonstrated a high degree of success in performing a wide range of HITs across MTurk (ie, master qualification). In addition, we added a simple qualification question to each HIT to detect spammers or irresponsible workers. Each HIT contained 4 questions, including the qualification question, and was assigned to 3 workers. Through different iterations of data labeling, workers were paid from US $0.03 to US $0.05 after completing each HIT. We collected the labels for the 122,135 tweets used in this study through different iterations, from April 2019 to November 2020. We regularly checked the quality of the submitted tasks to detect low-quality workers during each iteration and revoke their access to our tasks. Before the formal initiation of the process, we pilot-tested the design, response time, and complexity of the HITs in 2 iterations and revised the workflow accordingly. To label the data set provided in this paper, we used AMT as a crowdsourcing service and did not collect any personally identifiable information from the workers (participants) during the data labeling task. The experiments were carried out in accordance with relevant guidelines and the University of Calgary Conjoint Faculties Research Ethics Board’s regulations. We implemented the entire workflow in Python (Python Software Foundation) and used Boto3 Python Software Development Kit to connect to and work with AMT.

### Time Adjustment

**Figure 1:**
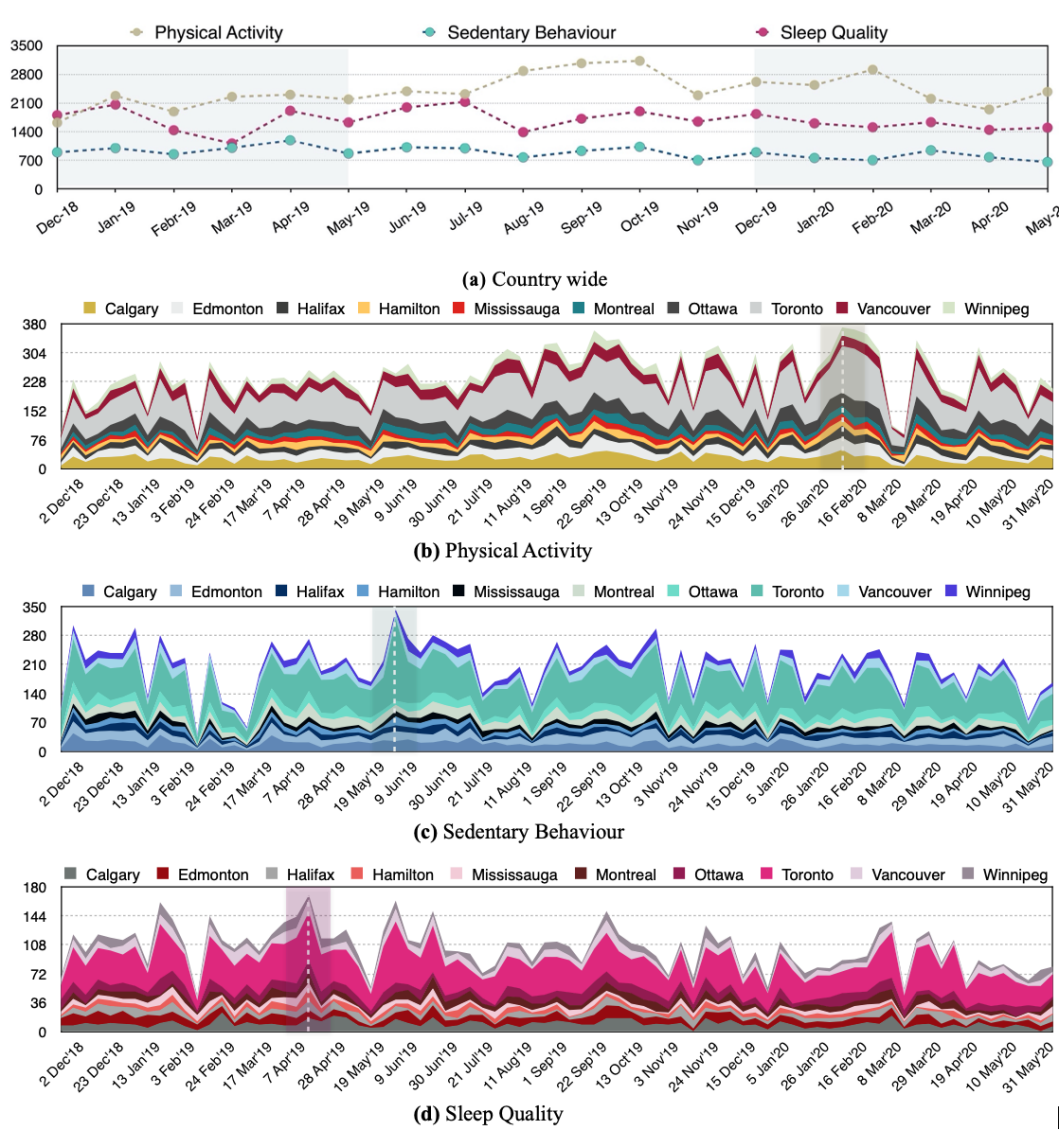
The temporal distribution of tweets for the Canadian data set. To make fair comparisons, we used data from December 1, 2018, to May 31, 2020, for these visualizations and removed data collected during the last 2 days of November 2018 and the first 2 weeks of June 2020.

The Twitter API returns the date and time that a tweet is published in the Universal Time Coordinated. To adjust this time zone based on each tweet’s location, we used the bounding box of coordinates, which enabled spatial mapping to tweets’ respective city locations, and used a time zone finder in Python. Given that daytime, month, and weekday can be influential factors in twitting about each of the PASS categories, and to better use the date-time data (%a %b %d %H:%M:%S %Y), we extracted weekday (a), month (b), and hour (H) fields and stored them as separate features. Figure 1A shows the temporal distribution of tweets for each of the PASS categories in the Canadian data set. Moreover, the stacked area charts presented in Figures 1B-1D detail the frequency of tweets for each of PASS categories for the top 10 Canadian cities.

### Location Inference

**Figure 2:**
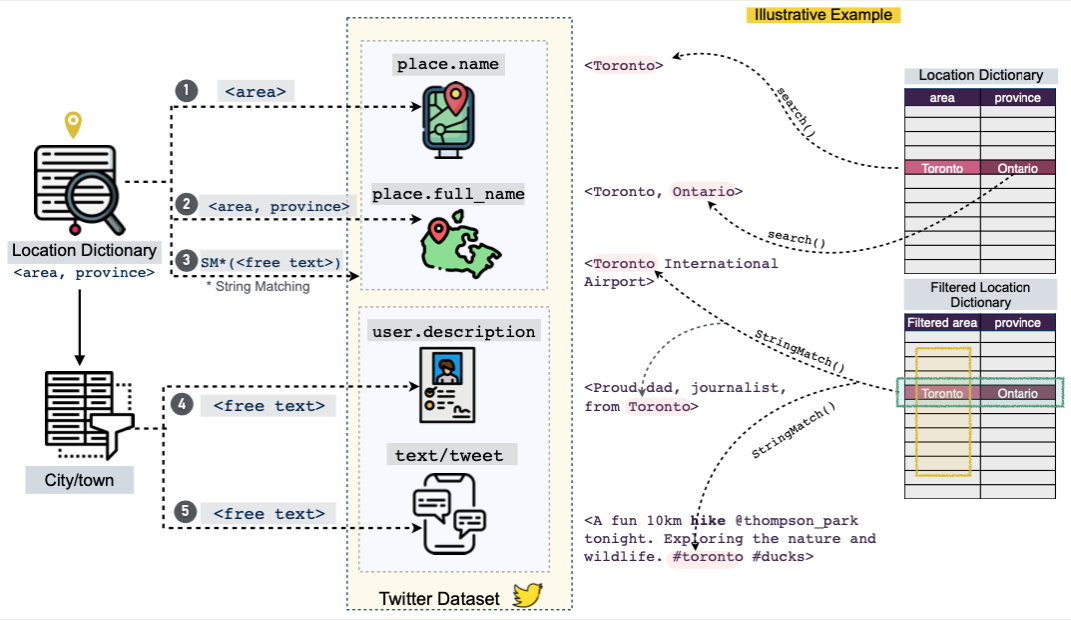
Five-step location inference process. The location dictionary is an external regional geographical metadata used to extract the exact locations of tweets. The area field in this process refers to regions at different scales such as city, region, municipality, town, township municipality, municipal district, dispersed rural community, village, or country.

The geospatial metadata provided by the Twitter API are derived from three main sources: (1) geotagged location, (2) profile location, and (3) mentioned location in the tweet text. Geotagged location can be defined by exact location (ie, device location) at the time of tweeting, by the assigned Twitter place (ie, at the neighborhood level), or both. Although the exact location field provides the highest level of precision, it is not a default setting, and only a small portion of users share their exact latitude and longitude (eg, only 1%-2% of tweets are geotagged [25]). Thus, to infer the location of each tweet in LPHEADA, we proposed and developed a 5-step process that uses tweet-neighborhood location (ie, *place.name* and *place.full_name*), profile information (ie, profile description and location), and tweet text to map Twitter’s geospatial metadata for each tweet to physical locations in the form of 〈c_i_,p_i_|s_i_〉, where *c* denotes the city of a tweet and *p*|*s* represent its corresponding province or state, respectively (Figure 2). To demonstrate the proposed process, we used the Canadian geographical names data set (ie, location dictionary) provided by the Geographical Names Board of Canada. Each geographical name provided by this data set is mapped to a province and is classified to a geographic area such as city, town, village, lake, administrative sector, or recreational center. As illustrated in Figure 2, for each *t_i_*, we first used a simple search function to map the place name field associated with each tweet to its corresponding *c_i_* in the location dictionary. If found, the corresponding province field *p_i_* was defined using equation 1:



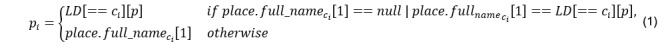



where 
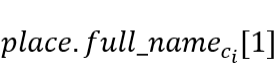
 denotes the second component of the field when the first component is *c_i_* (eg, Ontario in the illustrative example of Figure 2). This will detect geographical areas with the same names but in different provinces (eg, Leduc is a town or city in both Alberta and Quebec).

Steps 3-5 of the process deal with unstructured text objects that can come from all 3 sources of geospatial information. To extract the location information from these fields, we developed a string-matching function to detect the longest common substring between the unstructured text of the data set and the area field of the location dictionary (eg, first time boating in Lake Louise #AB is mapped to 〈Lake Louise,Alberta〉 instead of to 〈Louise,Quebec〉). To manage the complexity of information extraction from unstructured text, we only used a subset of areas listed in the location dictionary with high population density (eg, city, municipality, town, village, and country). Thus, we excluded areas classified as lake, mountain, river, bridge, or park.

### Demographic Attribute Inference

The demographic variables of age and gender and the information about the source of each tweet (eg, organizations vs real users) were not available within the data set collected from Twitter. We estimated these variables for each tweet using the M3inference package in Python [26], which uses a multimodal deep neural architecture for joint classification of age (binned into four groups: 18, 19-29, 30-39, and 40 years), gender, and information source of social media data. This approach uses 4 sources of information, namely, username, screen name, biography, and profile image of public profiles, to develop 2 separate pipelines for processing a profile image and each of the 3 text sources of information. The models provided in this package are trained on 14.53M, 2.61M, and 23.86M profiles for each of the gender, age, and organization categories, respectively.

## Results

### Data Records

#### Overview

LPHEADA is released in accordance with Twitter’s terms and conditions, and the developer’s agreement and policies [27], which prohibits the verbatim release of the collected tweets. However, releasing the tweet IDs is allowed. Data access requires a data use agreement between the data user and Twitter to govern the access and use of the licensed material returned by the Twitter API. Once approved, using the Tweet ID field, Twitter metadata can be *rehydrated* and downloaded as a JSON (JavaScript Object Notation) file to be mapped to other subsets of data provided in this study (eg, labels, location, time, and demographics). A detailed and demonstrative tutorial on rehydration of the data set using tweet IDs is described on the GitHub page of the data set [28].

**Figure 3:**
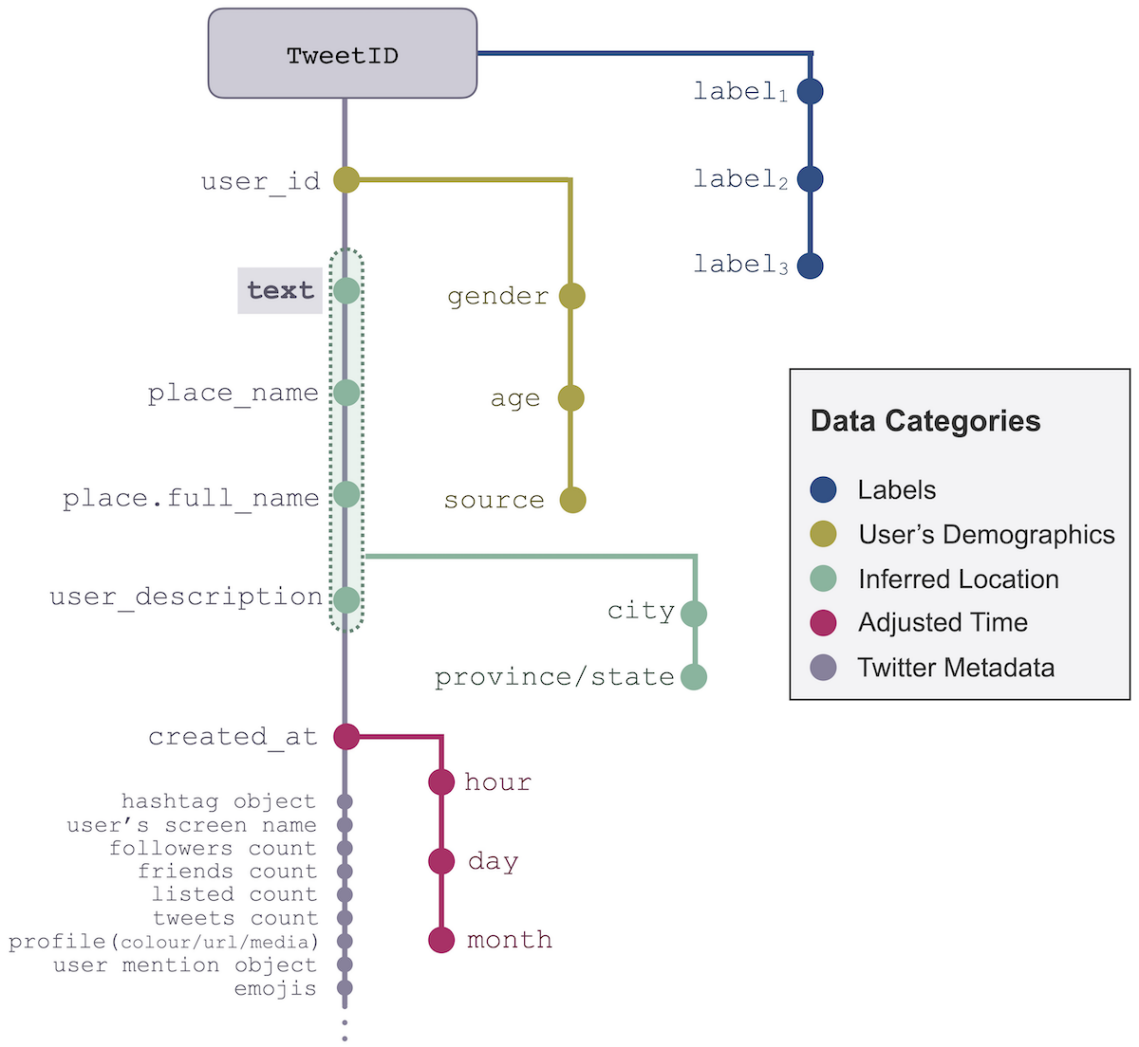
Overview of information tracking using TweetID. Each tweet or text is identified by a unique TweetID (provided by LPHEADA). This ID is mapped to metadata that includes user_id, place_name, place_full_name, user_description, and created_at. A total of 3 labels are provided for each TweetID that can be used for developing machine learning models. user_id was used to infer the demographics of each tweet, including gender, age range, and source. Adjusted time (month/day/hour) was extracted using created_at, and text, place_name, place_full_name, and user_description were used to identify the city and state or province mapped to each TweetID. LPHEADA: Labelled Digital Public Health Data Set.

LPHEADA comprises 366,405 labels for 122,135 unique tweets generated by 72,749 unique users between November 28, 2018, and June 19, 2020. This data set is organized into 12 subsets (3 PASS categories for each of the 4 countries). Table 1 provides the demographics of the data set, including the number of tweets per PASS category for each country, labels’ characteristics, and demographics characteristics of the users. Each unique tweet is assigned a unique integer, known as TweetID. Each ID is mapped to the core Twitter metadata and to 3 crowd-generated labels for binary and multi-class classification tasks. Figure 3 visualizes this hierarchy. As illustrated in this figure, for each labeled tweet, LPHEADA provides the following data categories.

**Table 1:** Characteristics of the data set.^a^

Variable						Canada (N=98,722)							United States (N=10,193)							United Kingdom (N=9154)							Australia (N=4067)				
						PA^b^		SQ^c^		SB^d^		PA			SQ		PA		PA			SQ		SB		PA			SQ		SB
Tweets, n (%)						48,576 (49.2)		32,779 (33.2)		17,367 (17.59)		5053 (49.57)			3065 (30.07)		2074 (20.35)		4076 (44.53)			3001 (32.78)		2077 (22.69)		2216 (54.49)			1312 (32.26)		539 (13.25)
**Labels**																															
	**Binary, n (%)**																														
			Yes		15,337 (31.57)		11,814 (36.04)		6514 (37.51)		1196 (23.67)			1032 (33.67)		731 (35.25)		1092 (26.79)			766 (25.52)		356 (17.14)		729 (32.9)			499 (38.03)		75 (13.91)	
			No		33,239 (68.43)		20,965 (63.96)		10,853 (62.49)		3857 (76.33)			2033 (66.33)		1343 (64.75)		2984 (73.21)			2235 (74.48)		1721 (82.86)		1487 (67.1)			813 (61.97)		469 (87.01)	
	**Multi-class, n (%)**																														
			YY		17,298 (35.61)		12,818 (39.1)		7174 (41.31)		1431 (28.32)			1150 (37.52)		831 (40.07)		1227 (30.1)			934 (31.12)		468 (22.53)		846 (38.18)			566 (43.14)		97 (18)	
			YN		6583 (13.55)		7720 (23.55)		1895 (10.91)		1445 (28.6)			1104 (36.02)		623 (30.04)		1037 (25.44)			1017 (33.89)		916 (44.1)		905 (40.84)			404 (30.79)		147 (27.27)	
			NY		4407 (9.07)		2242 (6.84)		1471 (8.47)		634 (12.55)			215 (7.01)		147 (7.09)		226 (5.54)			332 (11.06)		118 (5.68)		208 (9.39)			72 (5.49)		33 (6.12)	
			NN		19,622 (40.39)		9339 (28.49)		6502 (37.44)		1512 (29.92)			564 (18.4)		469 (22.61)		1559 (38.25)			694 (23.13)		572 (27.54)		233 (10.51)			258 (19.66)		258 (47.87)	
			NC		666 (1.37)		660 (2.01)		325 (1.87)		31 (0.61)			32 (1.04)		10 (0.48)		27 (0.66)			24 (0.8)		3 (0.14)		24 (1.08)			12 (0.91)		4 (0.74)	
**Users, n**																															
		Unique			22,601		16,984		11,490		4911			2994		2048		3810			2653		2002		1735			1004		517	
		Valid			21,772		14,919		10,912		3660			2614		1759		2614			2157		1840		1531			858		517	
**Gender, n (%)**																															
		Female			8471 (38.91)		7270 (48.73)		4486 (41.11)		1448 (39.56)			1537 (58.8)		860 (48.89)		911 (34.85)			1068 (49.51)		625 (33.97)		520 (33.96)			401 (46.74)		205 (39.65)	
		Male			13,301 (61.09)		7649 (51.27)		6426 (58.89)		2212 (60.44)			1077 (41.2)		899 (51.11)		1703 (65.15)			1089 (50.49)		1215 (66.03)		1011 (66.04)			457 (53.26)		312 (60.35)	
**Age range (years), n (%)**																															
		≤18			1772 (8.14)		2372 (15.9)		1361 (12.47)		490 (13.39)			518 (19.82)		318 (18.08)		136 (5.2)			248 (11.5)		136 (7.39)		114 (7.45)			132 (15.38)		68 (13.15)	
		19-29			5804 (26.66)		5575 (37.37)		3358 (30.77)		1469 (40.14)			1450 (55.47)		841 (47.81)		639 (24.45)			784 (36.35)		539 (29.29)		421 (27.5)			301 (35.08)		152 (29.4)	
		30-39			5609 (25.76)		3265 (21.88)		2605 (23.87)		761 (20.79)			363 (13.89)		302 (17.17)		705 (26.97)			540 (25.03)		486 (26.41)		421 (27.5)			209 (24.36)		139 (26.88)	
		≥40			8527 (39.16)		3707 (24.85)		3588 (32.88)		940 (25.68)			283 (10.83)		298 (16.94)		1137 (43.5)			585 (27.12)		659 (35.82)		575 (37.56)			216 (25.17)		158 (30.56)	
**Top 5 cities**																															
						Toronto		Toronto		Toronto		Los Angeles			Houston		Los Angeles		Cardiff			Leeds		Glasgow		Sydney			Sydney		Sydney
						Ottawa		Calgary		Calgary		Houston			Los Angeles		Houston		East Midlands			Sheffield		Manchester		Melbourne			Melbourne		Melbourne
						Calgary		Edmonton		Ottawa		Manhattan			San Antonio		Brooklyn		Bristol			London		Sheffield		Brisbane			Brisbane		Brisbane
						Vancouver		Ottawa		Edmonton		Chicago			Chicago		Chicago		Lambeth			Liverpool		Leeds		Perth			Adelaide		Perth
						Edmonton		Montréal		Vancouver		Florida			Brooklyn		Florida		Liverpool			Scotland		Edinburgh		Adelaide			Perth		Adelaide
**Source, n (%)**																															
				Organization		3109 (14.28)		727 (4.87)		1105 (10.13)		63 (1.72)			97 (3.71)		76 (4.32)		368 (14.08)			94 (4.36)		143 (7.77)		135 (8.82)			22 (2.56)		26 (5.0)
				Individual		18,663 (85.72)		14,192 (95.13)		9807 (89.87)		3391 (92.65)			2551 (97.59)		1638 (93.12)		2246 (85.92)			2063 (95.64)		1697 (92.23)		1396 (91.18)			836 (97.44)		491 (95.0)

^a^The 3 labels collected for each tweet were consolidated into a single label using majority voting. The discrepancy between the numbers of binary and multi-class labels is because of how majority voting calculates the truth label for each of these categories.

^b^PA: physical activity.

^c^SQ: sleep quality.

^d^SB: sedentary behavior.

#### Labels

Let *L* denote the set of *j* unique class labels*,* *t* represent the tweet text, and *w_k_* represent the *k^th^* worker who labeled the tweet, where *k* ∈ {1,2,3}. Each *l_j_* ∈ *L* is defined based on two conditions: whether the tweet is self-reported (*c*_1_ ∈ {0,1}) and whether the tweet reports a recent PASS experience (*c*_2_ ∈ {0,1}). For each PASS category across the 4 countries, the data set contains the following 2 subsets of labels for each tweet.

#### Multi-class Labels

In this subset, each tweet *t* is mapped to quadruple *L* = 〈*tweetID*,(*w*_1_, *l_j_*_1_),(*w*_2_,*l_j_*_2_),(*w*_3_,*l_j_*_3_)〉, where *j*=5 and each *l_j_* is defined based on the values of both *c*_1_ and *c*_2_ conditions and can be formulated as {11,10,01,00}. We also let workers choose a fifth option, called *unclear*, to ensure they do not give random labels to tasks that they are not confident of performing successfully. The 11, 10, 01, 00, and unclear labels correspond to the YY, YN, NY, NN, and NC labels, respectively, presented in Table 1.

#### Binary Labels

Each label *l_j_* in this subset is defined based on logical AND relationship between conditions *c*_1_ and *c*_2_. Thus, *l*_1_=1 if the tweet presents self-reported PASS surveillance and *l*_1_=0 otherwise. Like the multi-class category, each tweet is mapped to a quadruple, and there is a class called *unclear* (*l*_3_) with *j*=3. The binary labels did not directly come from the AMT workers and were generated by dichotomizing the collected labels.

### User’s Demographic Data

In the demographic data set, each tweet *t* is mapped to quadruple *D* = 〈*tweetID,a,g,o*〉, where *g* ∈{male, female} represents the gender of the user who posted the tweet, *a* ∈ {≤18,19-29,30-39,40≤} represents their age range, and *o* ∈ {0,1} shows the source of the tweet (ie, *o*=1 if the tweet was posted by an organization and *o*=0 otherwise). Table 2 shows the demographic distribution of the Canadian data set based on gender and age range of the unique users associated with tweet IDs. We can see that 79.24% (16,027/20,227) of female users in this data set are inferred to be aged <40 years, whereas this number for the male users is 57.55% (15,754/27,376). In addition, the most populated age category for female users across all PASS categories is 19-29 years, whereas this range for male users is ≥40 years. Excluding the (female, sleep quality) category, the age range ≤18 years, regardless of the user sex, is the least populated category across all PASS categories.

**Table 2:** Demographic information of users associated with the tweets originated in Canada (N=42,603).

		Age group, n (%)				
		Age≤18 years	Age 19-29 years	Age 30-39 years	Age≥40 years	Total
**Physical activity (n=21,772)**						
	Female	779 (3.58)	3160 (14.51)	2408 (11.06)	2124 (9.76)	8471 (38.91)
	Male	993 (4.56)	2704 (12.42)	3201 (14.7)	6403 (29.41)	13,301 (61.09)
**Sedentary behavior (n=10,912)**						
	Female	607 (5.56)	1822 (16.7)	1108 (10.15)	949 (8.7)	4486 (41.11)
	Male	754 (6.91)	1536 (14.08)	1497 (13.72)	2639 (24.18)	6426 (58.89)
**Sleep quality (n=14,919)**						
	Female	1267 (8.49)	3307 (22.17)	1569 (10.52)	1127 (7.55)	7270 (48.73)
	Male	1105 (7.41)	2268 (15.2)	1696 (11.37)	2580 (17.29)	7649 (51.27)

### Inferred Location Data

**Figure 4:**
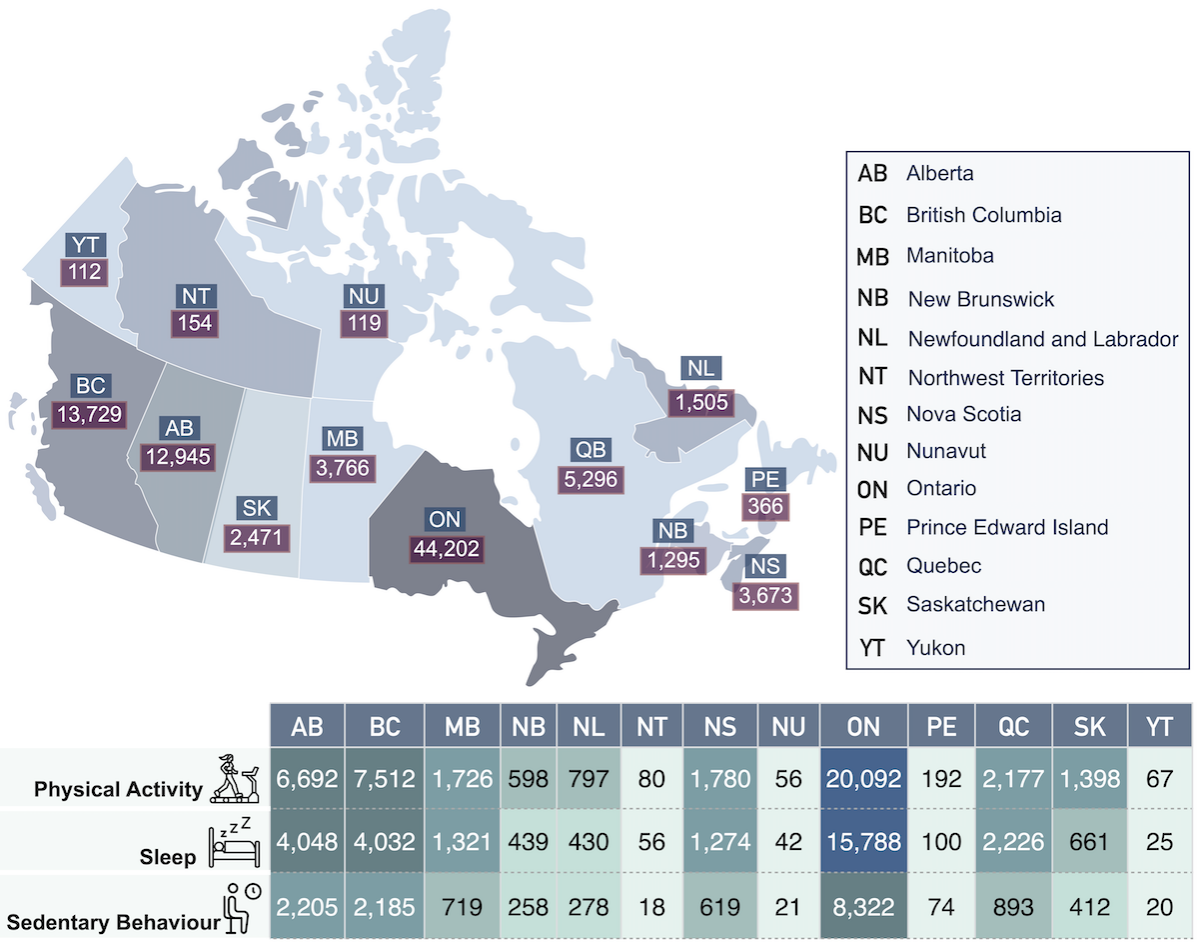
Geospatial details of the Canadian data set.

Each row of the location data set is presented in the form of *A* = 〈*tweetID,c,p*〉, where *c* and *p* denote the city and province or state associated with each tweet, respectively. Using the TweetID parameter, the location data can be mapped to other data sets, including labels, user demographics, time, and Twitter metadata. Each of *the c and p* variables is inferred based on the raw variables in Twitter metadata, including text, place objects, and user’s profile description (Figures 2 and 3). For example, Figure 4 shows the distribution of labeled tweets for each PASS category across Canadian provinces (ie, *p*). For the top 5 provinces, the overall size of the data set is directly proportional to the population size of each province. However, as only English tweets from Twitter users are included in the data set, LPHEADA represents only English-speaking Quebecor’s and Francophone Quebecor’s tweets in English, placing the province in fourth place. Moreover, with a lower population than British Columbia, Alberta had more sedentary behavior and sleep quality tweets and places in second place (Figure 4).

### Temporal Data

The temporal data set inferred from the *created_at* field of the Twitter metadata presents the adjusted time of each tweet based on the tweet’s location. Each row of this data set is presented in the form of *T* = 〈*tweetID,h,d,m*〉, where *h*, *d*, and *m* represent the hour, weekday, and month associated with each tweet, respectively. The *year* value does not need any adjustment and can be extracted directly from the original *created_at* field. For example, Figure 1 represents the frequency of tweets in each PASS category across Canada at the national (Figure 1A) and city levels (Figures 1B-1D). The highlighted area in Figure 1A demonstrates the data set’s temporal windows that can be used to compare different aspects of PASS surveillance between 2019 and 2020.

### Twitter Metadata

In addition to the inferred data records mentioned above, TweetIDs presented in LPHEADA can be used to retrieve Twitter metadata. This metadata, in addition to the tweet text, place object, time of the tweet, and user IDs, provides more details on the tweet and user objects, including the following.

#### User Object

This object comprises user’s screen name, description, follower count, friend count, listed count (ie, the number of public lists that the user is a member of), tweet count (ie, the number of tweets issued by the user), and profile characteristics (eg, image, color, and URLs).

#### Tweet Object

**Figure 5:**
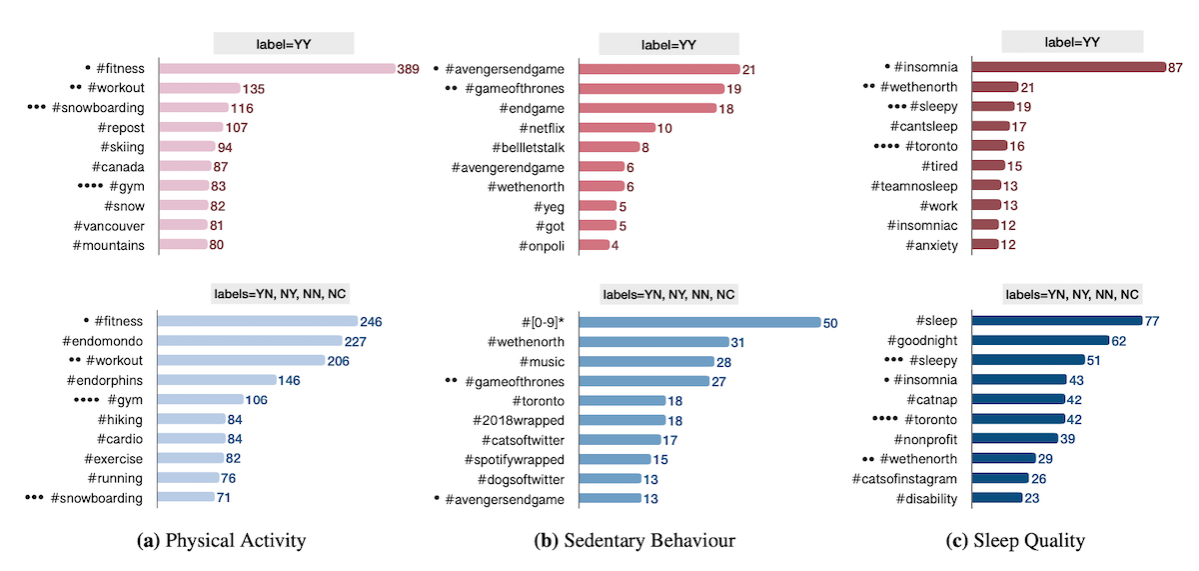
The distribution of the top 10 hashtags per label for each of the physical activity, sedentary behavior, and sleep quality categories. The label on top of each bar graph shows the class of tweets presented in the graph. For example, the YY category presents all tweets that are self-reported and describe a recent PASS experience. Similarly, YN presents all self-reported tweets but does not present a recent PASS experience. NC presents tweets with an unclear label. The number at the end of each bar presents the frequency of its corresponding hashtag. The intersections between 2 classes of labels for each PASS category are annotated using filled circles (•). Same hashtags are tagged with the same number of circles. This figure is based on all data collected from Canada, the United States, the United Kingdom, and Australia. PASS: physical activity, sedentary behavior, and sleep.

This object comprises hashtags mentioned in each tweet, emojis, user mentions, URLs, and media (eg, images and videos). For example, Figure 5 illustrates the distribution of the top 10 hashtags per label for each of the PASS categories in the entire data set. Hashtags are basically keywords or word strings prefixed with the symbol *#* that are used for categorizing and communicating tweets pertaining to the same topics. Although the high level of intersection between the hashtags of positive and negative classes in our data set makes this feature a less discriminating feature for the development of ML models (eg, annotated hashtags in Figure 5), this field can still be used by PASS-related advocacy campaigns on Twitter to brand their movement and open up their campaigns to users who need more information about the context [29]. As tagged tweets are easily archived and accessible, the hashtag field can be effectively leveraged to improve the public’s engagement in digital PHS discussions.

### General Release Notes

We have made our data set publicly available, along with instructions and Jupyter Notebooks [30,31] to illustrate the application of the data. All data and code (written in Python 3) used in this study are available through our GitHub repository [25]. We provide all necessary instructions, required libraries, and sample Jupyter Notebooks, allowing replicating our experiments and using the data set.

## Discussion

### Technical Validation

To verify the quality of crowd-generated labels and set a baseline for the data set, we conducted 4 studies. First, we used a series of statistical inference models to verify the quality of the labels provided in this data set. Second, we evaluated the semantic consistency between the data sets collected from the countries included in our repository. Third, we trained and tested 12 binary classifiers using the labels provided in the data set. Finally, to investigate the structural differences between all subsets of LPHEADA, we conducted linguistic and lexical analysis and visualized the results for further comparisons. Moreover, to address unseen technical issues of the data set, we provide a public issue tracker for handling bug reports, describing solutions to technical issues, data updates, and other issues and contributions.

### Methods of Label Agreement

To measure the consistency of labels generated by AMT workers, we calculated label consistency (*LC*) as the average entropy of the collected labels for each PASS category [32]. For each tweet *t_i_* ∈ *T_s_*, where *T_s_* denotes the set of all tweets related to surveillance category *s* ∈ {physical activity, sleep quality, sedentary behavior} and *s* ∈ {physical activity, sleep quality, sedentary behaviour}, *n_ij_* defines the number of answers given to the *j^th^* choice (*j* ∈ {1,2,3,4,5}), as we have 5 choices for each tweet), we calculate *LC* as follows:

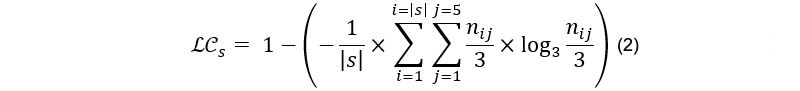



|*s*| denotes the size of the surveillance category *s*, and as we collect 3 labels for each tweet, the denominators in the entropy formula receive the constant value of 3*.* *LC* ranges from 0 to 1, and the values close to 0 show less consistency between workers’ input. After calculating *LC* for each PASS category, we had *LC*>0.52 for the multi-class labeling and *LC*>0.73 for the binary labeling task.

To consolidate the collected labels for each tweet, we used the majority voting (MV) technique (Table 1). Defining the estimated label as 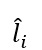, and the submitted label by worker *w* as *l_w_*, the MV approach for a binary labeling task assigns 1 to 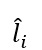 if 
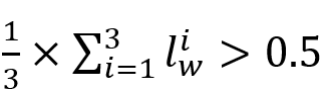
 and assigns 0 if otherwise. The discrepancy between the number of binary and multi-class labels presented in Table 1 is caused by the way that MV approach calculates the truth label for each of these categories. In addition to MV, there are models, such as those by David and Skene [33] and Raykar et al [34] and the generative model of labels, abilities, and difficulties [35], that incorporate the error rate of annotators (workers), task complexity, and context-sensitive features into the inference process and can be used to predict truth labels from crowd-labeled data.

### Semantic Consistency

To validate the semantic consistency of the data sets collected from different countries, we transformed the data set of each PASS category into a semantic space of low dimensionality using latent semantic analysis. For the vector presentation of each data set, to capture high-level semantics of the text, we ran the PASS category of each country through a pretrained word2vec embedding model. This model contains 300-dimensional vectors of 3 million words and phrases trained on 100 billion words from a Google News data set. The resulting 300-dimensional vectors were then averaged for each tweet. For each *tweet* *T* composed of words 〈*w*_1_,*w*_2_,...,*w_n_*〉, with 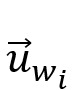 defining the embedding of *w_i_*, the embedding of *tweet* *T* can be calculated as:

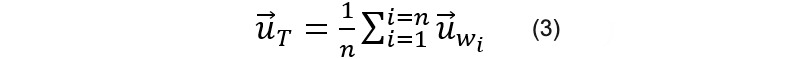



**Figure 6:**
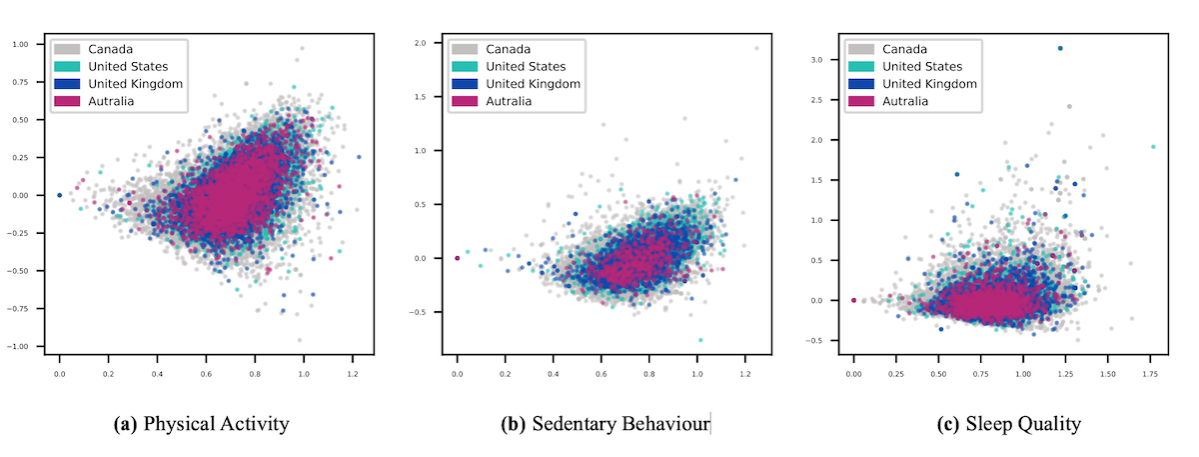
Scatter plots of the first 2 dimensions of latent semantic analysis performed on physical activity, sedentary behavior, and sleep categories, and classified based on the geographic source of the data.

We then applied truncated singular value decomposition on the new vectorized data set and kept the top 2 dimensions of the data set containing the most variance (eg, those directions in vector space of the data set that contain more information). The scatterplots presented in Figure 6 illustrate our data sets in a 2-dimensional latent semantic space. The high level of overlap between the data sets of each PASS category indicates that the data from different countries cover similar semantic space, but the space is scaled differently based on the size of the data sets.

Moreover, to further investigate the internal consistency of the data sets presented in this paper, we trained 3 convolutional neural network multi-class classifiers (ie, 1 for each PASS category) to classify tweets into Canada, US, Australia, or UK classes. Given the highly imbalanced distribution of the classes in our data set due to the highly unequal number of samples from each country, we used the average precision (AP) metric to measure the discrimination ability of our predictive models. The poor performance of these classifiers (*AP_PA_*, 37%; *AP_SB_*, 32%; *AP_SQ_*, 31%) in detecting each tweet’s country implies a high level of semantic and syntactic cohesion among the 4 countries in our data set.

### Classification of PASS Categories

For the PASS classification experiment, we used a standard convolutional neural network classifier with 1 layer of convolution with global max-pooling on top of a word2vec embedding trained on 100 billion words of Google News. The vectors have a dimensionality of 300 and were trained using the continuous bag-of-words architecture [36]. We used the binary labels of the data set to train and evaluate the model on each of 12 data sets provided in LPHEADA. Owing to the imbalanced distribution of binary labels across these data sets (Table 1), in addition to precision, recall, F1, and area under the curve (AUC) scores, we used AP to measure the weighted mean of precision at different thresholds to make the score robust to heterogeneous and imbalanced class distributions. Like AUC score, AP is a model-wide and threshold-free evaluation metric. However, for imbalance class distributions with the negatives outnumbering the positives, AP is more informative than AUC, as it mainly evaluates the fraction of true positive samples among positive predictions and is more robust to the relationship between false-positive and false-negative rates [37]. As shown in Table 3, for each of the Canada, US, and UK data sets, we find a steady increase in the overall performance of the classifier as the size of the data set increase (ie, |PA|>|SQ|>|SB|). Interestingly, the UK data set achieves the highest performance for the PA category among all the countries.

**Table 3:** Binary classification of tweets using bidirectional long short-term memory.^a^

Metrics		Country (%)			
		Canada	United States	United Kingdom	Australia
**Physical activity**					
	Precision	78	75	80	65
	Recall	78	76	81	68
	F1	78	76	80	64
	AUC_ROC_^b^	83	76	84	64
	AP^c^	81	75	81	66
**Sedentary behavior**					
	Precision	73	62	79	74
	Recall	73	64	83	86
	F1	73	62	79	80
	AUC_ROC_	76	62	63	57
	AP	78	65	66	66
**Sleep quality**					
	Precision	76	70	73	61
	Recall	76	70	76	60
	F1	75	70	73	60
	AUC_ROC_	81	70	66	63
	AP	83	72	71	64

^a^The same classifier is used to classify the data from different countries.

^b^AUC*_ROC_*: area under the receiver operating characteristic curve.

^c^AP: average precision.

### Linguistic Properties

**Figure 7:**
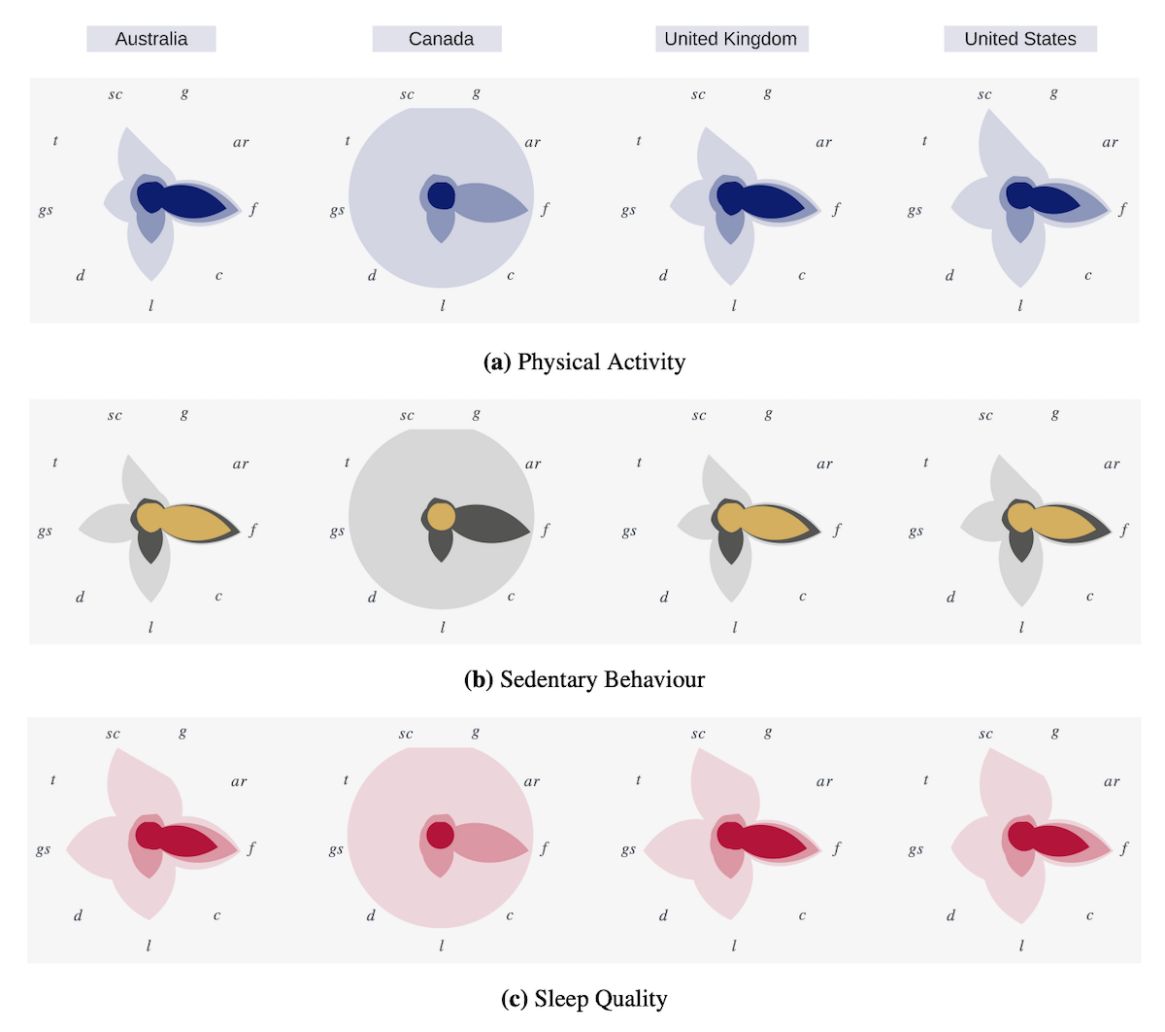
Linguistic comparisons across different countries. *ar*: automated readability index; *c*: Coleman-Liau Index; *d*: Dale-Chall readability; *f*: Flesch-Kincaid grade level; *g*: Gunning Fog index; *gs*: grammar score; *l*: Linsear Write readability; *sc*: sentence count; *t*: text readability consensus.

To understand and validate the linguistic properties of each data set, we measured and visually compared the following metrics for each PASS category grouped by country: (1) sentence count, (2) grammar score (ie, number of grammar errors), (3) the average number of syllables per word and the average sentence length (ie, Flesch-Kincaid Grade Level index [38]), (4) the average number of words per sentence and the percentage of words with 3 or more syllables (ie, Gunning Fog index [39]), (5) a combination of average sentence length and percentage of difficult words (ie, Dale-Chall readability [40]), (6) sentence length and number of polysyllables (ie, Linsear Write readability [40]), (7) number of characters (ie, Coleman-Liau Index [40,41]), (8) the average number of characters per word and number of words per sentence (ie, automated readability index [38]), and (9) the text standard score based on number of sentences, words, syllables, and characters in each tweet (ie, text readability consensus). Figure 7 illustrates these comparisons based on the minimum (red), average (pink), and maximum (light pink) values of each feature. Although all data sets have similar behavior in terms of each feature’s minimum value, the Canadian data set has a lower score for the average number of syllables per word and the average sentence length for all PASS categories. Interestingly, the sleep quality data set, compared with other PASS categories, has a higher value for the maximum number of *grammar errors* and *sentence count* metrics, whereas all data sets show the same behavior for the minimum and the average values of these metrics. These location-specific linguistic characteristics should be considered when using these data sets to train and evaluate PASS surveillance ML models. For example, a model trained on the Canadian data set may not present some linguistic features of a data set that originated in Australia and vice versa.

### Limitations

Several limitations should be noted. First, we collected our data set using Twitter’s free streaming API, which returns a random sample of about only 1% of global public tweets produced at a given time. However, our data set spans 19 months of tweets posted by users from 4 English-speaking countries, which provides enough diversity and coverage for conducting retrospective and comparative digital public PASS studies. Moreover, the search terms used to filter the data set could have impacted the topics included in our data set, which may influence the generalizability of the results derived from this data set. To address this and to ensure the lists of context-sensitive terms for filtering all the PASS categories are comprehensive enough, we used domain-specific ontologies, WordNet [42], and NLP techniques (eg, topic modeling, language modeling, and lexical analysis) to detect latent word patterns to identify PASS-related contexts in unstructured text.

Despite these limitations, the curated, validated [43], and labeled data set provided in this paper will allow researchers and practitioners to delve into different aspects of digital PASS surveillance by developing ML, NLP, and exploratory models. We believe that the novelty and comprehensiveness of this data set will help the development, evaluation, and deployment of digital PASS surveillance systems, and it will be an invaluable resource for both public health researchers and practitioners.

## Appendix

Multimedia Appendix 1 A complete list of the regular expressions and filtering words for each PASS domain.

## Abbreviations

AMT: Amazon Mechanical Turk
AP: average precision
API: application programming interface
AUC: area under the curve
DPHS: digital public health surveillance
HIT: human intelligence task
LC: label consistency
LPHEADA: Labelled Digital Public Health Dataset
ML: machine learning
MV: majority voting
NLP: natural language processing
PASS: physical activity, sedentary behavior, and sleep
PHS: public health surveillance
