# Supplementary material for: Physical Activity, Sedentary Behavior, and Sleep on Twitter: Multicountry and Fully Labeled Public Data Set for Digital Public Health Surveillance Research
Source: JMIR Public Health Surveill. 2022 Feb 14;8(2):e32355. doi: 10.2196/32355 (PMC8887637; doi:10.2196/32355)
Supplement: Multimedia Appendix 1 [file publichealth_v8i2e32355_app1.pdf]

Sleep Keywords

	Sleep	
C_1	C_2	C_3
sleep	fall	chronic
slept	felt	paralysis
asleep	deprive	apnea
awake	hour(s)	consciousness
sleepless	schedule	asthma
wake	night	provigil
bed	tonight	hypersomnia
nap	depth	stress
sleepiness	work	trouble
rest	wakefulness	clinic
heaviness	little	treatment
	(o) clock	suffer
	heaviness	quality
	day	eszopiclone
	alarm	ramelton
	week	rexall
	circadian	zolpidem
	rhythm	pill
	early	pediatric
	cannot	melatonin
	can't	zaleplon
	lack	anxiety
	light	disease
	short	aid
	catnap	pressure
	caffeine	sick
	slumber	pain
		headache
		mediation
		insomnia
		problem
		modafinil
		nightmare
		sopor
		disorder

Physical Activity Keywords

C$_1$
football, tennis, baseball, run[ing, ran], basketball, rugby, bball, hockey, volleyball, cricket, soccer, badminton, golf, ski[ing, ed], swim(ming), climb[ed, ing], jogg[ing, ed], step, aerobic, jazzercise, karate, yoga, treadmill, gymnastic, golf, skate, workout, zumba, parkour, boxercise, exercise, forward[-, λ]roll, isometric, dance[ed, ing], warm[-, λ]down, sit[-, λ]up, cardio, press[-, λ]up, stair[-, λ]climbing, pull[-, λ]up, stretch[ed, ing], stomach[-, λ]crunch, t'ai chi, hunt, salsa, power[-, λ]walking, kayak, isotonic, bicycle[ing], hapkido, hike[ed, ing], canoeing, kickball, vball, VB, lacrosse, bike, squash, wrestl[ed, ing], martial, snowboard, cycle, dodge[-, λ]ball, frisbee, dumbbell, lift[ing], elliptical, muscle, bowling, boogie boarding, body[-, λ]boarding, sprinting, softball, parachute, snowshoeing, weight[-, λ]belt, triceps curl bar, incline bench, [upper+lower] body, glute bridge, reverse crunch, inverted rows, dead[-, λ]lift, squat, cable shrugs, foam[-, λ]roll, bench reps, power[-, λ]lifting, curl ball, overhead press, leg press, pec-deck, jump crossbar, mountain, uphill, [BMX+RPM+Spine], jumping jack, weight lift[ing], stair[-, λ]treadmill, ergometer, rope skip[ping], rowing, kettle[-, λ]bell, going [up+down] [stairs+mountain], ski machine, slide board, slimnastics, pilates, exercise ball, airdyne, calisthenics, nadi[-, λ]sodhana, surya namaskar, ballet, treadmill desk, archery, billiard, broomball, tether[-, λ]ball, curling, hang gliding, handball, horseback riding, jai alai, paddle[-, λ]ball, racquetball, skydiving, shuffleboard, base jumping, bungee jumping, ping pong, trampoline, wallyball, hammer throw, discus throw, shot puts (throw), hurdle, nordic walk(ing), springboard, kayaking, sailing, windsurfing, jet skiing, paddle boarding, water[-, λ]polo, toboggan[ing], bobsled[ding], bobsleigh, luge, kickboxing
C$_2$
today, tomorrow, hour, week, day, time, yesterday, tonight, $[1..9]^+[0..9]^*$hr, morning, afternoon, tonight, [Sunday-Saturday], $[1..9]^+[0..9]^*$min/sec, [one..ten]min/hr/hour/sec, fast, slow, $[1..9]^+[0..9]^*$[km+kilo+mile], ago, month, interval, just, now, routine, plan, daily, weekend, $[1..9]^+[0..9]^*$lb(s), sets, $[1..9]^+[0..9]^*$mph, every(λ)day, hr[s], will
C$_3$
went, finish[ed, ing], complet[ed, ing], end[ed, ing], did, do, go, play[ed, ing], class, train, fat, calorie[s], weight, feel, felt, burn, going to, wellness, press, hit, max[ed] out], great, hurt, gassed, try, tried, try[ing], was doing, light, record, teach[ing], coach[ing], my, want, complete [d, ing], strength, gym, workout, my—today's

Sedentary Behavior Behaviour

C$_1$
PlayStation, X[-, λ]box, GameCube, TV, television, DVD, video, computer, Nintendo, season, episode, chapter, YouTube, Nintendogs, Minecraft, Kindle, puzzle, crossword, Twitter, Facebook, Instagram, movie, game, GameCube, Internet, YouTube, iTunes, Skype, WhatsApp, chess, Tumblr, music<br>watch, play, read, drive, drove, sit[ting], sat, study[ing], studied, draw[ing], drew, sketch[ed, ing], lay[ing], laid, stream[ing], gaming, research, play[ed, ing], sewing, ride, rode, coding, programming, writing, editing, texting, reading, revising, standing, painting<br>bus, traffic, homework, class, library, computer, tablet, iPad, phone, desk job
C$_2$
day, today, night, one sitting, $[1..9]^+[0..9]^*$hr, morning, afternoon, [Sunday-Saturday], $[1..9]^+[0..9]^*$min/sec, [one..ten]min/hr/hour/sec, hrs straight, evening
